# Beyond the Basics: Best Practices in Scrotal Ultrasound for the Infertile Male

**DOI:** 10.1590/S1677-5538.IBJU.2025.9920

**Published:** 2025-10-20

**Authors:** Francesco Lotti

**Affiliations:** 1 University of Florence Department of Experimental and Clinical Biomedical Sciences "Mario Serio" Florence Italy Department of Experimental and Clinical Biomedical Sciences "Mario Serio", University of Florence, Florence, Italy; 2 University Hospital Careggi (AOUC) Female Endocrinology and Gender Incongruence Unit Florence Italy Andrology, Female Endocrinology and Gender Incongruence Unit, University Hospital Careggi (AOUC), Florence, Italy

## INTRODUCTION

Infertility affects up to 12% of men (1-3). Despite scientific advances, especially in sperm biology and genetics, its etiology is still unknown in half of the cases (1, 2). To fill this gap, the imaging of the male genital tract (MGT) has progressively expanded to improve diagnosis, allowing for the complete evaluation of the infertile male when medical history, physical examination, semen analysis, and blood parameters do not provide sufficient information for adequate management (2). The use of MGT imaging to investigate infertility is recommended by the European Academy of Andrology (EAA) (3-7), the European Society of Urogenital Radiology (ESUR) (8), the European Association of Urology (EAU) (9), and the American Urological Association/American Society for Reproductive Medicine (AUA/ASRM) (10). In addition, MGT imaging is useful for assessing male general health, improving the characterization of scrotal and pelvic pain, inflammation, or masses of the MGT organs (1-3, 6, 11-14).

In the evaluation of the infertile male, color-Doppler ultrasound (CDUS) represents the gold-standard method to investigate the scrotal (2, 4, 6, 7) and prostate-vesicular (2, 13-17) regions. US is a simple, rapid, and harmless diagnostic tool and, among imaging techniques, is the least expensive (2, 7). Scrotal US can assess (i) features related to testicular damage, associated with non-obstructive oligo-/azoo-spermia, astheno- and/or terato-zoospermia, (ii) abnormalities of the epididymis and/or vas deferens, suggesting partial or complete obstruction of the proximal seminal tract, and (iii) varicocele (2,6-8). Prostate-vesicular US can investigate features related to obstructive oligo-/azoo-spermia and/or low seminal volume and pH (2, 5, 6, 8, 16, 17), as well as characteristics suggestive of prostate and seminal vesicles inflammation or malignancy (2, 5, 13-15, 17).

This Expert Opinion critically addresses the role of scrotal CDUS in the evaluation of the infertile male, with implications for both reproductive and general health, according to evidence-based studies. In addition, it reports on Standard Operating Procedures (SOPs) to perform scrotal CDUS properly.

### Scrotal CDUS

Scrotal CDUS is useful to assess (i) reproductive health, (ii) scrotal pain, (iii) masses, and (iv) trauma (2, 7, 11, 18).

Concerning reproductive health, CDUS can detect abnormalities in the size, echotexture, and vascularization of the testes, which are associated with sperm abnormalities and low testosterone levels (2, 7, 8). Furthermore, it provides information on epididymis and vas deferens alterations associated with sperm abnormalities (2, 7, 8). Finally, it allows the detection and staging of varicocele, which could negatively influence sperm parameters (2, 8, 19).

As for scrotal pain/soreness, CDUS can detect abnormalities in the size and echotexture of the testes or epididymis. These abnormalities are associated with hypervascularization, suggesting inflammation (orchitis or epididymitis), or absent testicular vascularization, suggestive of testicular torsion or infarction (2, 7, 11). Furthermore, scrotal CDUS can detect varicoceles or inguinal/scrotal hernias, which may be associated with discomfort, a sense of heaviness, or pain (2, 7, 13, 19).

CDUS also plays a key role in the study of testicular and extratesticular masses, characterizing them as benign or malignant with good accuracy, although without providing diagnostic certainty. It is also involved in the investigation of risk factors for testicular cancer (TC), such as cryptorchidism and diffuse microlithiasis (2, 7, 11). Finally, CDUS is useful to evaluate scrotal trauma (18).

**Figure 1:**
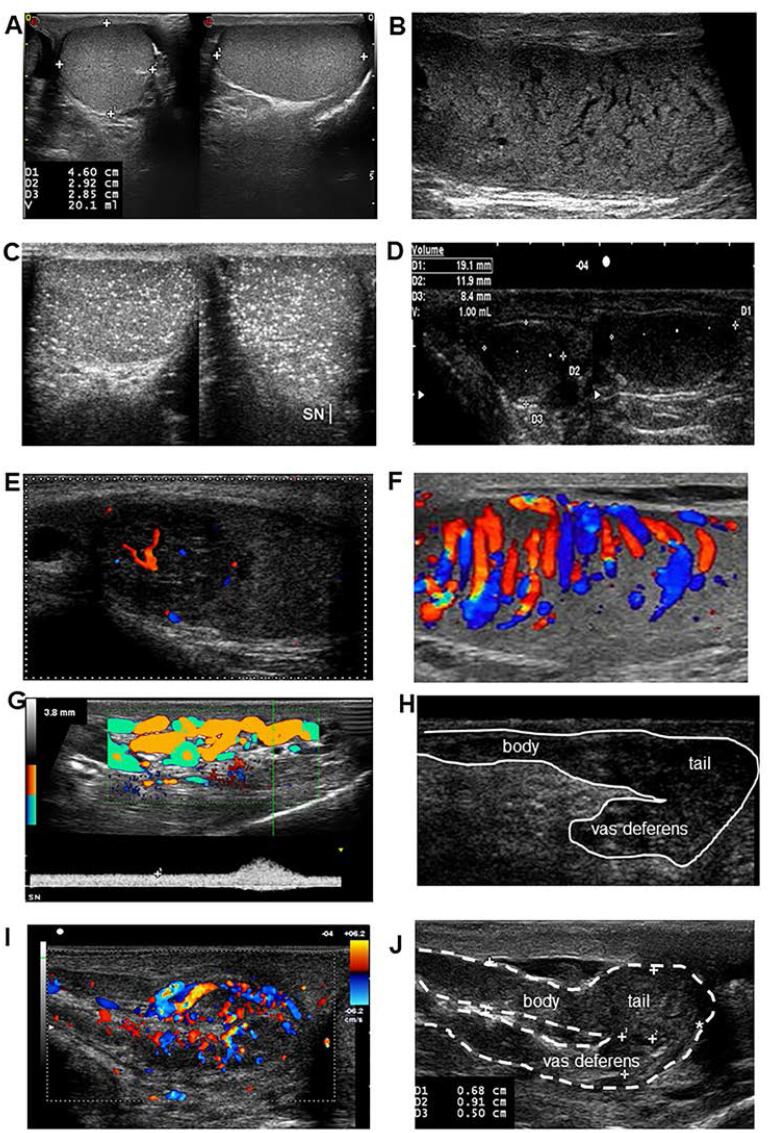
A) normal testis (normal volume, homogeneous, normoechoic); B) testicular inhomogeneity ("hypoechoic reticulation"); C) diffuse "starry sky" microlithiasis; D) cryptorchid testis (hypotrophic, inhomogeneous, hypoechoic); E) vascularized testicular nodule (seminoma), F) orchitis; G) grade 4 varicocele; H) agenesis of the vas deferens; I) acute epididymitis (body and tail); J) dilated epididymis (body and tail). Adapted from (2, 7).

The EAA recently developed SOPs for CDUS evaluation of the scrotal organs (Table-1) based on a multicenter consensus (4,6,7), and published evidence-based "normative" CDUS parameters derived from healthy, fertile men (3, 4, 6) (Table-2). More recently, the ESUR produced recommendations on the role of scrotal imaging in evaluating male infertility (8). Below and in Table 3, the main scrotal CDUS parameters are reported to investigate male reproductive and general health. Figure-1 shows some normal and pathological CDUS findings.

**Table 1:** EAA Standard Operating Procedures (SOPs) to assess scrotal CDUS.

Testis
**Testicular volume**
Evaluate the three maximum diameters of each testis (anterior-posterior [height] and transverse [width] diameters in transverse scan; longitudinal diameter [length] in longitudinal scan)
Calculate testicular volume using the ellipsoid formula (length x height x width x 0.52)
**Testicular homogeneity**
Use a four point-Likert scale: 0. homogeneity 1. mild (grade 1) inhomogeneity [presence of small hypoechoic foci/thin hypoechoic striae] 2. moderate (grade 2) inhomogeneity [presence of thick hypoechoic striae – "zebra-like appearance"] 3. severe (grade 3) inhomogeneity [diffuse inhomogeneity with "reticulation"/"geographical map" appearance])
**Testicular echogenicity**
Use a three point-Likert scale: 0.normoechoic 1.mainly hypoechoic 2.mainly hyperechoic
**Calcifications and microlithiasis**
Macrocalcifications: calcifications with a size > 3 mm
Microcalcifications: small (1-3 mm) bright echogenic foci with no acoustic shadowing
Microlithiasis: presence of ≥ 5 microcalcifications in a single US scan, classified as 1.limited, 2.‘clusters’ or 3.diffuse (‘starry sky’ appearance). Report localization in the upper, middle and lower third of the testis
**Testicular nodules**
Evaluate the three diameters and characteristis (0.cystic; 1. mixed; 2.solid), shape (0.regular; 1.irregular), homogeneity (0. homogeneous; 1.inhomogeneous), echogenicity (0.normal echogenicity; 1.mainly hypoechoic; 2.mainly hyperechoic), calcifications and/or cysts (0.absent; 1.present) and vascularization (0.absent, 1.peripheral, 2.intranodular)
**Testicular vascularization**
Qualitative assessment: normal, reduced, enhanced (in the entire testis and/or focal areas); compare the two testes
Quantitative assessment*: evaluate arterial PSV (or acceleration, RI and PI) in the testicular artery -in the spermatic cord, 2 cm before the gonadal hilum- and the intratesticular arteries (recurrent rami of the centripetal arteries).
**Other findings**
Evaluate and measure **dilated rete testis**
Evaluate and measure **parenchimal cysts**
Evaluate and measure **testis appendices**
Evaluate and measure **extratesticular calcifications** (including scrotoliths).
Evaluate and measure **hydrocele** (three diametes and volume); use convex probe when bulky.
**Epididymis and vas deferens**
Evaluate the CDUS features of the three epididymal segments (head, body and tail) and vas deferens
**Size (diameters)**
Head: measure the longitudinal diameter from the top to the base of the triangle
Body and tail: measure the anterior-posterior diameters in a single longitudinal scan (if possible including the proximal vas deferens)
Vas deferens: evaluate presence or absence. Measure the anterior-posterior diameter (if possible in the same longitudinal scan with epididymal body and tail)
**Homogeneity/inhomogeneity**
Report it as a dummy variable (0. homogeneous; 1. inhomogeneous),
**Echogenicity**
Use a three-point Likert scale (0. normal echogenicity; 1. mainly hypoechoic; 2. mainly hyperechoic)
**Vascularization**
Qualitative assessment: normal, reduced, enhanced; compare the two epididymes
Quantitative assessment*: evaluate arterial PSV(or acceleration, RI and PI) at the level of the head (branch of the testicular artery) and of the tail (branch of the the deferential artery)
**Other findings**
Evaluate the presence of **nodules** (in the same way of "testicular nodules")
Evaluate the presence and number of **cysts**
Evaluate and measure epididymal **calcifications**
Evaluate and measure epididymal **appendices**
**Pampiniform plexus/varicocele**
1. Measure the largest vein, irrespective of location, with the patient standing, at rest, bilaterally. CDUS varicocele is defined in presence of venous vessels > 3 mm at rest, with retrograde venous flow detected at least during Valsalva manouvre.
2. Evaluate the extension of the largest vein to the funicular region, upper or lower pole of the testis.
3. Evaluate the presence of a retrograde venous flow in the patient standing, at rest, using CDUS, and classify it as a dummy variable (0.absent or intermittent/fluctuating during spontaneous breath; 1.continuous).
4. Then evaluate the variation of venous flow during Valsalva manouvre. -if basal retrograde venous flow in the patient standing, at rest, is absent, report if there is vascular enhancement during Valsalva manouvre (if yes: varicocele grade 1-3 according to extension of the largest vein to the funicular region, upper or lower pole of the testis, respectively – see below *EAA classification of varicocele*) -if basal retrograde venous flow in the patient standing, at rest, is present, perform Valsalva manouvre and report if there vascular enhancement (grade 4) or not (grade 5) – see below *EAA classification of varicocele*).
Use Sarteschi et al./Liguori et al. classifications for grading varicocele (7, 8).
"Severe" varicocele: venous vessels dilation (> 3 mm) characterized by a continuous venous reflux at rest, increasing or not during a Valsalva manoeuvre (consistent with grade 4 and 5 of Sarteschi et al./Liguori et al. classifications)
Subclinical varicocele: venous reflux detected by CDUS but not clinically evident
**EAA classification of varicocele.** -grade 1: venous vessels dilation (> 3 mm) at rest at the funicular region with retrograde venous flow absent/intermittent at rest and enhanced during Valsalva manouvre. -grade 2: venous vessels dilation (> 3 mm) at rest at the upper pole of the testis with retrograde venous flow absent/intermittent at rest and enhanced during Valsalva manouvre. -grade 3: venous vessels dilation (> 3 mm) at rest at the lower pole of the testis with retrograde venous flow absent/intermittent at rest and enhanced during Valsalva manouvre. -grade 4: venous vessels dilation (> 3 mm) at rest (irrespective of location, but usually extending to the peritesticular region) with retrograde venous flow *continuous* at rest and enhanced during Valsalva manouvre. Possible testicular hypotrophy. -grade 5: venous vessels dilation (> 3 mm) at rest (irrespective of location, but usually extending to the peritesticular region) with retrograde venous flow *continuous* at rest and not increasing during Valsalva manouvre. Possible intratesticular varices and/or testicular hypotrophy.

The EAA SOPs are derived and adapted from the EAA scrotal US study (4). PSV, peak systolic velocity; RI, resistive index; PI, pulsatility index.

* So far, testis and epididymis vascular "quantitative" assessment is not routinely recommended.

**Table 2:** EAA CDUS reference ranges and classifications for the scrotal organs and thresholds suggesting CDUS abnormalities.

		EAA CDUS reference ranges and classifications for the scrotal organs	Thresholds suggesting CDUS abnormalities of the scrotal organs
**Testis**			
	Mean TV (ellipsoid)	17 ± 4 mL	Mean testis hypotrophy: < 12 mL
	Right TV	Range: 12 – 26 mL	Right testis hypotrhophy: < 12 mL
	Left TV	Range: 11 – 24 mL	Left testis hypotrhophy: < 11 mL
	Testicular inhomogeneity (TI): classification	0. Homogeneity	Any testicular inhomogeneity: pathologic
		1. Mild inhomogeneity (presence of small hypoechoic foci/thin hypoechoic striae)	
		2. Moderate inhomogeneity (presence of thick hypoechoic striae-"zebra-like appearance")	
		3. Severe inhomogeneity (diffuse TI with "reticulation"/"geographical map" appearance)	
	Testicular microlithiasis (TML)	Normal:<5 microcalcifications per field of view	TML: ≥ 5 microcalcifications per field of view
	Testicular vascularization	Normal: ome color-Doppler spots with discrete distribution Norma PSV of: -testicular artery: 3 – 11 cm/s -intratesticular artery: 3.7 – 7 cm/s	Pathologic: -Diffuse testicular hyperemia: a)diffuse: suggestive of orchitis or, more rarely, diffuse testicular hematological neoplasms b)in a testicular nodule: suspected tumor -Absence of testicular vascularization: a) diffuse: suspected torsion; b) limited, in a cuneiform hypoechoic area: suspected lobular infarction
**Epididymis and vas deferens**			
	Epididymal head	Range: 7 - 11.5 mm (with no cysts) Range: 7 – 12 mm (with cysts)	Dilated >12 mm: likely inflammation or distal obtruction
	Epididymal body	Range: 2.5 - 5 mm	Dilated > 5 mm: likely inflammation or distal obtruction
	Epididymal tail	Range: 4 - 6 mm	Dilated > 6 mm: likely inflammation or distal obtruction
	Vas deferens	Range: 2.3 - 4.5 mm	Dilated > 4.5 mm: likely distal obstruction
	Vascularization	Normal: discrete color-Doppler spots following the deferential artery route	Pathologic: Diffuse hyperemia or one or more segments: current inflammation
**Varicocele**		Normal: absent (venous vessels < 3 mm with no basal or provoked reflux)	Pathologic: varicocele: Venous vessels > 3 mm at rest, irrespective of location, with retrograde venous flow detected at least during Valsalva manouvre, with grading according to Sarteschi et al. /Liguori et al. See EAA classification (7) and ESUR recommendations on varicocele (19).

TV = testicular volume; PSV = peak systolic velocity; EAA = European Academy of Andrology; ESUR = European Society of Urogenital Radiology; CDUS = color-Doppler ultrasound.

**Table 3:** Scrotal color-Doppler ultrasound (CDUS) and reproductive and general health: what to investigate and why.

Main scrotal CDUS parameters to evaluate		Why to evaluate
**Testis**		
	Volume	-Positive association with sperm parameters and testosterone; negative association with FSH and LH and unconventional sperm parameters (e.g., sperm DNA fragmentation) -Bilateral very small (<4 mL) [and hard, with elevated gonadotropins] testes suggestive of Klinefelter syndrome -Bilateral small [and soft, with low gonadotropin levels] testes suggestive of hypogonadotropic hypogonadism -A normal volume (with normal FSH) does not exclude NOA
	Echotexture	-Testicular inhomogeneity: negative association with sperm parameters and testosterone levels -Rete testis dilation: suggestive of post-testicular obstruction -Multiple hypoechoic micronodules in Klinefelter syndrome: suggestive of Leydig cell hyperplasia islets
	Nodular lesions/masses	Solid or mixed nodules, vascularized: suggestive of cancer
	Microlithiasis	-Association with testicular cancer (especially in men with "additional risk factors" or with "starry sky microlithiasis"): perform annual follow-up up to 55 years of age. -Possible association with infertility (debated)
	Localization	-Cryptorchidism or history of cryptorchidism/orchidopexy: negative association with sperm parameters and testosterone levels; increased risk of testicular cancer: annual follow-up up to 55 years of cryptorchid and contralateral testis.
	Vascularization	-Absent: a) diffusely: testicular torsion (especially in men with pain); b) localized: possible lobular infarction -Diffuse hyperemia: sign of ongoing inflammation (orchitis) or, more rarely, of diffuse hematological neoplasms (leukemia in children, lymphoma in elderly men). -All cases: possible transient or permanent negative effect on sperm parameters (and possibly on testosterone levels)
	Varicocele	-Negative association with sperm parameters (and, sometimes, with testosterone levels), especially for high grades (4 and 5) -Association with male infertility debated
**Epididymis**		
	Dilatation	- Suggestive for post-testicular (sub)obstruction (at the level of the (i) epididymis [if vas deferens with regular size], (ii) vas deferens [including CBAVD or CUAVD] or (iii) prostate [evaluate the prostate-vesicular region with US]) with possible negative effect on sperm parameters -Suggestive of previous or ongoing inflammation, with possible negative effect on sperm parameters -Only overt bilateral epididymal dilation (suggested, but not proven, with US) is associated with OA
	Hyperemia	- Sign of ongoing inflammation (epididymitis), with possible transient or permanent negative effect on sperm parameters
	Absence	-Associated with CBAVD with OA -Associated with CUAVD with normal or altered sperm parameters (see "vas deferens")
**Vas deferens**		
	Dilation	-Suggestive of downstream (sub)obstruction, including (i) obstruction of the retroperineal vas deferens [possibly evaluable with MRI] or (ii) vasectomy or (iii) surgical sequelae of repair of inguinal hernia or, (iv) rarely, absence of the distal portion of the deferens] or (v) at the level of the prostate [evaluate the prostate-vesicular region with US to investigate EDO -including MPC-]) with possible negative effect on sperm parameters
	Absence	-CBAVD associated with OA -CUAVD: normal or altered sperm parameters -extend the investigation to the prostate-vesicular region to study the SV (bilateral absence in 50% of CBAVD subjects; ipsilateral absence in 90% of CUAVD subjects) and to the abdomen to study the kidneys (frequent ipsilateral absence in CUAVD men, rare unilateral absence in CBAVD men) and consider genetic counseling (especially for CBAVD, evaluate *CFTR* gene mutation).

NOA = non-obstructive azoospermia; OA = obstructive azoospermia; CBAVD = congenital bilateral absence of vas deferens; CUAVD = congenital bilateral absence of vas deferens; EDO = ejaculatory duct obstruction; MPC = midline prostatic cyst; SV = seminal vesicle; CFTR = Cystic Fibrosis Transmembrane Conductance Regulator. Adapted from (8).

### Testicular volume

Testicular volume (TV) evaluation is critical in investigating the infertile male because it generally mirrors the testicular function. TV correlates positively with all conventional sperm parameters and testosterone levels, and negatively with FSH and LH levels (2, 4, 6, 7), as well as with unconventional semen parameters (e.g., sperm DNA fragmentation, chromatin compactness, mitochondrial membrane potential, phosphatidylserine externalization, apoptotic M540 bodies) (2, 7). TV reflects not only seminal and hormonal status but also previous or current testicular or systemic disorders (2, 7).

TV is usually estimated in clinical practice with the Prader orchidometer, which offers a good surrogate of the real TV, and correlates positively with the US-TV in both fertile and infertile subjects (2, 4, 6, 7). However, the evaluation of TV by the US is more accurate. It is necessary when the physical examination is not informative, such as in the presence of a large hydrocele, inguinal cryptorchidism, small testis, or epididymis enlargement (2, 7).

US-TV can be calculated using different mathematical formulas (e.g., ellipsoid, Lambert's, and Hansen's), starting from the measurements of length (d1), width (d2), and height (d3) of the testis (2, 7, 8). The EAA (4, 6) and ESUR (8) support the ellipsoid formula ( TV=d1×d2×d3×0.52 ), which correlates better with the Prader orchidometer-TV and is easier to use in clinical practice since US consoles automatically calculate it.

According to the EAA, the average TV in healthy, fertile men is 17±4 mL, and is significantly lower in infertile subjects (4, 6). The lower reference limit of US-TV for right and left testes in fertile males is 12 and 11 mL, respectively, evidence-based thresholds defining "testicular hypotrophy" (4,6). Very small (<4 mL) and hard testes, associated with elevated gonadotropin levels, suggest Klinefelter syndrome (2, 8). Small, soft testes associated with low gonadotropin levels suggest hypogonadotropic hypogonadism (2, 8). However, a normal TV does not exclude non-obstructive azoospermia (NOA), since patients with maturation arrest often have normal TV (2, 8).

### Testicular echotexture

The normal adult testis is characterized by a homogeneous granular echotexture, consisting of uniformly distributed medium-level echoes (homogeneous and normoechoic testis) (2, 4, 6, 7). The alteration of the echotexture, and in particular testicular inhomogeneity (TI), is often related to testicular damage, abnormal sperm parameters, and low testosterone levels (2, 7, 8, 20, 21).

TI investigation is critical because, unlike TV, it cannot be assessed clinically and can only be evaluated with the US. TI is characterized by the presence of hypoechoic parenchymal striae (expression of a greater representation of the interlobular septa, usually not visible, and periseptal tubular atrophy), which give a "zebra-like appearance" to the testis, or, in more severe cases, by the presence of a hypoechoic "reticulation" or a "geographic map" appearance (2, 7, 8).

On histology, TI reflects parenchymal atrophy and fibrosis (2, 7). TI has been detected in numerous conditions associated with male infertility, including cryptorchidism and acquired testicular damage (2, 7, 8). Furthermore, TI is frequently observed in Klinefelter syndrome, often characterized by hypoechoic micronodules and the expression of islets of Leydig cell hyperplasia (2, 7, 8). TI has historically been classified on a 5-point scale (2, 7, 8) and, recently, by the EAA on a 4-point scale (4,6), where higher scores suggest more severe testicular damage. As a corollary, the testis echotexture alteration also includes rete testis dilation, which suggests post-testicular obstruction (2, 8).

### Testicular microlithiasis

Testicular microlithiasis (TML) is a US diagnosis, defined as ≥5 microcalcifications (bright hyperechoic spots <3 mm with no acoustic shadowing) per visual field (2, 7, 8). Its association with infertility and TC is widely debated. Regarding infertility, although some studies reported a higher prevalence of TML in infertile compared with fertile men, the TML-infertility association is not fully recognized (2, 7, 8). Regarding TC, recent meta-analyses supported a significant association with TML. However, literature reviews report that TML is not an independent risk factor but is associated with TC when "additional risk factors" are present (7, 8, 11). The ESUR guidelines recommend annual US follow-up up to age 55 in patients with TML and "additional risk factors" (personal/family history of TC, cryptorchidism, orchidopexy, testicular atrophy, infertility) and in men with diffuse TML ("starry sky") (8).

### Cryptorchidism

Cryptorchidism is the absence of at least one testis in the scrotum (2,7,8,11,22). Its prevalence is 30% in premature newborns, 3% in full-term newborns, 1% in children at the third month of life (2, 7, 8, 11, 22), and, notably, almost 10% in males with severe oligozoospermia (23). The undescended testis is unilateral in 90% of cases. Approximately 80% of undescended testis are located within the inguinal canal, 5-16% in the abdomen, and are rarely ectopic (2, 7, 8, 11, 22).

Cryptorchidism is associated with an increased risk of infertility and TC (2, 7, 8, 11, 22). Infertility has been reported in ~10% of men with unilateral and almost 40% of men with bilateral cryptorchidism (22). The risk of TC is 3-6-fold higher than in the general population (22). TC usually develops in the undescended testis; however, 20% of TC develop in the contralateral descended testis (2, 7, 8, 11, 22).

The ESUR recently recommended performing testicular US in men with a history of cryptorchidism due to the increased risk of infertility and TC (8). The US plays a key role in cancer detection and/or in the follow-up of the cryptorchid and contralateral testis, and an annual US follow-up is recommended up to age 55 (8). In addition, it is recommended to perform scrotal/inguinal US in adult men with a nonpalpable testis (8). If the US is equivocal, inguinal/abdominal MRI or surgical exploration is advocated (8). In the US, the cryptorchid testis is often hypotrophic, non-homogeneous, hypoechoic, and with calcifications. Nodular lesions may be present and should be managed according to available guidelines (2, 7-9, 11, 24).

### Testicular lesions

Testicular lesions represent a clinical and US challenge. They can be detected incidentally during male infertility screening and/or when a subject complains of the detection of a scrotal lump, discomfort/sense of heaviness, or, rarely, scrotal pain (2, 7, 11). When dealing with large, hard, palpable nodules, management is primarily clinical and requires testis CDUS to confirm that they are solid, vascularized lesions suggestive of malignancy (2, 7, 11). However, when CDUS characteristics are uncertain, or when lesions are nonpalpable, "multiparametric US", which includes grey-scale and color-Doppler US combined with contrast-enhanced US (CEUS) and sonoelastography, improves their characterization to differentiate benign and malignant lesions (7, 11). This is very important, since testicular lesions are frequent, TC are the most common neoplasms in young adults (which are those of reproductive age and include most of infertile men), and the accurate evaluation of a testicular lesion is essential to define its correct management: testicular salvage and US follow-up or orchiectomy (2, 7, 11). The main clinical and multiparametric US characteristics of benign and malignant testicular lesions are reported in detail elsewhere (7, 11). Recently, ESUR published recommendations on the impact of US on the management of nonpalpable testicular lesions (24).

### Testicular vascularization

Testicular vascularization plays a key role in the diagnosis of (i) orchitis, where it appears diffusely increased, (b) malignancy, generally hypervascularized, (c) testicular torsion or infarction, where the vascularization is absent in a diffuse or scattered manner, respectively, and (iv) scrotal trauma (2, 7, 8, 11, 18). All the above-mentioned conditions can be associated with sperm abnormalities (2, 7, 8). Recently, the EAA reported a standardization of the measurement of testicular vascular parameters and their reference ranges in healthy, fertile subjects (4, 6).

### Varicocele

Varicocele is an abnormal dilatation of the pampiniform plexus characterized by retrograde venous flow (2, 8, 13, 19). The prevalence in men with primary infertility is ~35% (2, 8, 13, 19). Similar data have been found in healthy, fertile men (4, 6). Several studies report abnormal sperm parameters in infertile subjects with varicocele (2). However, 75% of subjects with varicocele have normal semen parameters (2). Therefore, the impact of varicocele on couple fertility is still debated, but it seems modest, and international scientific societies support varicocele correction only in highly selected cases (2, 6, 8). Physical examination has a lower accuracy in detecting varicocele compared to CDUS (2, 8, 19). CDUS is useful to assess varicocele, mainly (i) when physical examination is inconclusive or unreliable, (ii) to confirm and better classify a clinical varicocele, and (iii) to detect post-operative recurrence/persistence (2, 8, 19). Recently, ESUR reported recommendations for the standardization of CDUS in varicocele (19), and in agreement, EAA has produced a shared classification of varicocele (7). ESUR and EAA underline the importance of a standardized examination and provide diagnostic criteria (6-8, 19) (Tables 1 and 2).

### Epididymis and vas deferens

Scrotal US is the gold-standard imaging tool to investigate the epididymis and vas deferens (2, 7, 8). Their evaluation is critical, especially to distinguish OA and NOA in specific cases. In particular, the congenital bilateral absence of the vas deferens (CBAVD) and the bilateral complete obstruction of the epididymis are associated with OA (2, 7, 8). Furthermore, CDUS is useful to investigate epididymitis in subjects with scrotal pain (2, 7, 8). Recently, the EAA reported a standardization of measurements and identified reference ranges and normative thresholds for the size of the epididymal segments (head, body, tail < 12, 5, and 6 mm, respectively), proximal vas deferens (<4.5 mm) (4, 6) and deferential ampulla (<6 mm) (5,6) (Table-2) and related vascular parameters (4, 6).

### Vas deferens

The US detection of CBAVD places a specific diagnosis of OA (2,7,8). CBAVD is present in 1-2% of infertile men and in 4-17% of azoospermic men (2,7,8,25). Since CBAVD is often associated with seminal vesicle (SV) agenesis, azoospermia is frequently linked to low seminal volume and pH. Therefore, US examination should be extended to the prostate-vesicular region (2, 7, 8, 25) (Table-3).

Since CBAVD is usually associated with the mutation of the CFTR (Cystic Fibrosis Transmembrane Conductance Regulator) gene, genetic counseling is recommended in affected individuals (2, 7, 8, 25) (Table-3). Men with CBAVD usually have normal TV and testicular function. Therefore, if they want to achieve a pregnancy, surgical sperm retrieval is indicated (2, 7).

Scrotal US can also detect congenital unilateral absence of a vas deferens (CUAVD). This condition is present in 1% of infertile men. However, men with CUAVD may show normal semen parameters and be fertile (2, 7, 8, 25). Since CUAVD is frequently associated with agenesis of the ipsilateral SV, affected subjects may present low seminal volume and pH, and the US examination should be extended to the prostate-vesicular region (2, 7, 8, 25) (Table-3). Since CUAVD is frequently associated with ipsilateral renal agenesis (rare in patients with CBAVD), the US examination should also be extended to the abdominal region (2, 7, 8) (Table-3). Finally, although CUAVD is not usually associated with mutations in the CFTR gene, genetic counseling is prudent (7, 8). In cases of CAVD, epididymis may be present and dilated, often with tubular ectasia, or it may be partially absent (2, 7, 25). In both cases, the head of the epididymis is always detectable and can be dilated or small (2).

### Epididymis

Scrotal US plays a key role in investigating abnormalities in the size, echotexture, and vascularization of the epididymis, which, when considered alone or in combination, can suggest different diagnoses (2, 7, 8, 25). In subjects with scrotal pain or prostatitis-like symptoms, epididymal dilation associated with hypervascularization suggests inflammation (2, 7, 8, 11). A dilated epididymis associated with echotexture abnormalities may also represent the outcome of a previous infection/inflammation in pauci-/a-symptomatic patients (2, 7, 8). In subjects with obstructive oligo-/azoo-spermia, epididymal dilatation with tubular ectasia may suggest, as an indirect sign, post-testicular obstruction, at the level of the (i) epididymis, (ii) vas deferens, or (iii) prostate (2, 7, 8, 12, 16, 17), the latter to be investigated by extending the US examination to the prostate-vesicular region (5, 6, 8, 16, 17). Current or previous inflammation of the epididymis and/or its obstruction has been associated with sperm abnormalities (2,7,8,12). Only proven bilateral epididymal complete obstruction can diagnose proximal OA. However, so far, the US can only suggest, but not demonstrate, the presence of epididymal complete obstruction (8). Scrotal US also allows the evaluation of epididymal nodules, often represented by cysts, with no proven role in OA, or rarely by tumors (2, 11).

## CONCLUSIONS

Scrotal CDUS is useful for investigating and managing the infertile male, addressing both reproductive and general health. The use of SOPs, report standardization, and knowledge of normative parameters to distinguish normal and pathologic CDUS features and attribute them with correct clinical meaning are decisive for performing a correct US and benefiting from it for diagnostic and management purposes.

## Data Availability

All data generated or analysed during this study are
